# Influenza Pandemic Periodicity, Virus Recycling, and the Art of Risk Assessment

**DOI:** 10.3201/eid1201.051013

**Published:** 2006-01

**Authors:** Walter R. Dowdle

**Affiliations:** * The Task Force for Child Survival and Development, Decatur, Georgia, USA

**Keywords:** Influenza, pandemic, risk assessment, swine influenza

## Abstract

Influenza pandemic risk assessment is an uncertain art. The theory that influenza A virus pandemics occur every 10 to 11 years and seroarcheologic evidence of virus recycling set the stage in early 1976 for risk assessment and risk management of the Fort Dix, New Jersey, swine influenza outbreak. Additional data and passage of time proved the theory untenable. Much has been learned about influenza A virus and its natural history since 1976, but the exact conditions that lead to the emergence of a pandemic strain are still unknown. Current avian influenza events parallel those of swine influenza in 1976 but on a larger and more complex scale. Pre- and postpandemic risk assessment and risk management are continuous but separate public health functions.

## Abstract 2

Conditions that lead to influenza pandemics are not fully understood.

"I am sure that what any of us do, we will be criticized either for doing too much or for doing too little…. If an epidemic does not occur, we will be glad. If it does, then I hope we can say… that we have done everything and made every preparation possible to do the best job within the limits of available scientific knowledge and administrative procedure."

**—US Surgeon General Leroy Burney, Meeting of the Association of State and Territorial Health Officers, August 28, 1957** (*1*)

In 1941, on the eve of US entry into World War II, concern about a repeat of the 1918 influenza pandemic and its effect on armed forces led the US military to establish the Commission on Influenza (later combined with other commissions to become the present Armed Forces Epidemiological Board) and place high priority on developing a vaccine (*2*). Pandemic influenza did not materialize, but the vaccine did. The first successful large-scale influenza vaccine field trials were completed in 1943 (*3*). In 1947, failure of the vaccine to provide protection against the epidemic influenza type A antigenic variant confirmed concerns of vaccine obsolescence and led to the term "antigenic shift" (*4*) and designation of the 1947 FM1 strain by the Commission on Influenza as subgroup A´ on the basis of the hemagglutination inhibition (HI) test.

In May 1957, with reports of a potential influenza pandemic in the Far East, risk assessment responsibilities of the Commission on Influenza were clear. The Department of Defense influenza immunization policy of 1954 mandated quick formulation and provision of a new vaccine. The Public Health Service had no such official policy and found risk assessment to be a challenging process that relied heavily on international sources for surveillance and the Influenza Commission for advice. "There was no indication it would become a killer of the 1918 variety, but neither was there positive assurance it would not" (*1*). Risk management was contingent on evidence of "continued low mortality" or "increased virulence" (*1*). The consensus by late June was probable sporadic local occurrences during the summer with an epidemic during fall or winter that would bring only a relatively small increase in deaths. On August 28, the Surgeon General recommended immunization through established physician-patient channels. The watchword was to "alert but not alarm" the public and to generate interest in receiving the vaccine (*1*).

The 1957 Asian virus pandemic simultaneously increased knowledge of influenza pandemics and the complexity of future pandemic risk assessments. The pandemic had appeared exactly 10 years after appearance of the A´ virus, which suggested pandemic periodicity (*5*). Preexisting HI antibodies to the 1957 A2/Asian virus in sera collected before the pandemic were reported for some persons >75 years of age, which suggested that human influenza viruses were recycling (*6*).

In July 1968, with reports of influenza epidemics again appearing in the Far East, the US Military Commission on Influenza quickly obtained strains and recommended a new vaccine (*2*). Risk assessment by the Public Health Service this time around was a much simpler process. Annual vaccine recommendations to physicians for persons at high risk for death or severe complications were by now a matter of course. The need for a new vaccine was apparent (*7*), but early reports consistently described the disease as mild (*8*), and the US epidemic was over before the A2/Hong Kong virus was recognized as an antigenic shift (*9**,**10*).

The 1968 pandemic added to the complexities of risk assessment. The new subtype had appeared, right on time, 11 years after the 1957 Asian pandemic and replaced the dominant influenza A2/Asian virus subtype, as had the viruses of 1947 and 1957. Further, most persons >85 years of age had preexisting antibodies to the 1968 virus, which suggested that the hemagglutinin of this virus, as well as that of the 1957 virus, had appeared previously in the human population (*11*).

In 1976, speculation was rife that a new pandemic strain was due in a few years. The concept of 10- to 11-year influenza A virus pandemic patterns, with disappearance of the predecessor virus, seemed entrenched in the influenza literature. Previous influenza pandemics had occurred in 1968, before that in 1957, and before that in 1947; carrying the logic further, pandemics also occurred in 1929, 1918, 1900, and 1890 (*12*). The concept was supported by the World Health Organization (WHO) classification scheme, which implied that 4 influenza A subtypes had occurred in humans since 1933. In addition, seroarcheologic findings had been interpreted as evidence that the swine virus had last appeared in 1918, the Hong Kong virus (now designated H3) in 1900, and the Asian (H2) virus in 1890, not exactly 10–11 years apart, but in the same order (*13**,**14*). To some, the next pandemic virus in the sequence was the swine virus of 1918 (*13*).

On February 13, 1976, the New York Times published a guest editorial to remind the public and policy makers that influenza pandemics had marked the end of every decade—every 11 years—since the 1940s. The editorial urged accelerated pandemic planning and coordinated vaccine research (*15*).

## Risk Assessment in 1976

Coincidentally, on February 14, 1976, the day after the Times editorial was published, the Center for Disease Control (CDC) hosted an emergency meeting with the US Army, Food and Drug Administration, National Institutes of Health, and New Jersey State Health Department to assess the isolation of swine influenza virus from the late January outbreak at Fort Dix, New Jersey (*16*). Information was insufficient at the time to assess whether the swine influenza virus outbreak was a unique event in susceptible young recruits or the beginning of a pandemic, but the isolation of a predicted potential pandemic strain almost on schedule did not go unnoticed.

On March 10, the Army provided data to the US Advisory Committee on Immunization Practices that confirmed person-to-person transmission of swine influenza virus (*17*). The single swine influenza death loomed large, although most cases were mild. No one at the advisory committee meeting equated the disease potential of this virus with 1918, but the association of swine influenza virus with the most devastating pandemic in memory was widely speculated in the news media. Slightly more than a month after the outbreak, no evidence suggested that a pandemic would or would not occur; a situation such as the Fort Dix outbreak had never been encountered. On March 18, the action memo from the Assistant Secretary of Health to the Secretary, Department of Health, Education, and Welfare stated that "severe epidemics, or pandemics, of influenza occur at approximately 10-year intervals" and publicly linked swine flu with the pandemic of 1918 (*18*).

When WHO convened a meeting of consultants in Geneva on April 7 (*19*), 3 months had passed without evidence of further swine virus transmission anywhere in the world. The swine A/New Jersey strain had not replaced the current A/Victoria strain, which continued to circulate at Fort Dix well into February, and no evidence of swine/Victoria virus reassortants had been seen. Theories of what might happen were being overtaken by the realities of what was happening. The Fort Dix outbreak was beginning to look like an isolated event.

A report from the United Kingdom on the behavior of swine influenza virus in infected human volunteers would not appear in Nature for some weeks (*20*), but in April early rumors circulated that swine A/New Jersey virus was more infectious than classic swine virus but that the symptoms were mild to moderate. The report added little to risk assessment; the findings were consistent with events seen in the outbreak. But an accompanying editorial in Nature summarized the UK and likely European view, which urged caution in vaccine stockpiling and immunization programs and continuing assessment, "until the shape of things to come can be seen more clearly" (*21*).

Beginning in April and continuing into May, a group of US investigators used the Delphi technique to obtain an expert risk assessment with minimal bias (*22*). The 15 participating scientists and epidemiologists concluded that if swine influenza virus were to circulate in the United States, the epidemic would more likely resemble those of 1957 and 1968 than that of 1918. The probability of further swine influenza virus outbreaks was estimated at 0.10.

On August 1, a series of news reports began to appear on a fatal respiratory illness among American Legionnaires attending a convention in Philadelphia (*18**,**23*). Wide but inappropriate speculation that the cause of these unprecedented deaths might be swine influenza, accompanied by equally unprecedented national publicity, precluded further opportunity for rational risk assessment.

## Theory of Predictable Pandemics

Unknowingly, at the same time as the Fort Dix outbreak, the Working Group on Pandemic Influenza met in Rougemont, Switzerland, on January 26–28. Issues addressed included the growing body of evidence linking the origin of antigenic shift to animal reservoirs of influenza viruses (*24*), the questionable validity of predictable patterns of pandemic periodicity, and the appropriate classification of the 1947 strain (*25*).

When the 1947 epidemic occurred, only 13 years had passed since the first influenza virus was isolated. Available scientific knowledge was limited. No precedent existed for defining a pandemic strain or distinguishing antigenic shift (a complete change) from antigenic drift (point mutations resulting in accumulated amino acid changes). The 1957 Asian pandemic virus provided the first evidence of a true antigenic shift. The hemagglutinin and neuraminidase surface antigens were totally different from those of their 1956 predecessors. The 1968 Hong Kong pandemic virus provided evidence that antigenic shift can occur in the hemagglutinin independent of the neuraminidase, which was largely unchanged. The 1947 strain failed to meet the definition of an antigenic shift.

The 1971 revision of the system of nomenclature (*26*) recognized the independence of the 2 surface antigens and linked antigenic shifts with influenza A virus subtypes but further confounded the issue by designating the 1947 strain as a subtype for historical reasons. In the 1980 revision, which combined antigenically closely related subtypes regardless of source of isolation (*27*), the previous hemagglutinin designations of swine (Hsw1) and human H0 and H1 subtypes became H1N1, ending a misclassification of the 1947 strain that had endured for >30 years.

Thus, counting 1890, a total of 4 recognized pandemics have been separated by 28 years (1918), 39 years (1957), and 11 years (1968). Excluding the emergence of the H1N1 virus in 1977, an additional 38 years have elapsed since the last pandemic. No predictable pattern of pandemic periodicity exists.

## Pandemic Virus Recycling

In 1935, high levels of antibodies to the newly isolated influenza viruses from humans (*28*) and swine (*29*) were commonly seen among persons >10 years of age, which suggested that the 1918–1920 pandemic had been caused by the same or a closely related virus. The birth dates associated with the peak prevalence of swine virus (H1) antibodies did not change in sera collected 12, 17, or 20 years later (*30*). The seroarcheologic findings were validated in 1999 by sequencing the HA gene recovered from persons who died of influenza during the pandemic (*31*). Thus, swine (H1) virus was present from 1918 to 1920 and left a lifelong immunologic imprint on most persons who were <25 years of age at the time. Validation of the H1 seroarcheologic model allowed reexamination of earlier reports of preexisting H2 (1957) and H3 (1968) antibodies in sera collected from elderly persons before the respective pandemics (*32*).

### Serologic Findings Linking 1890 with H3

After 1957, preexisting H2 antibodies were not commonly observed. Three laboratories reported preexisting H2 antibodies among the elderly, while 3 other laboratories found no orientation of H2 antibody toward any particular age group. Further, peak antibody prevalence from the 2 primary laboratories (*6**,**30*) differed by nearly 8 years. The lack of agreement among investigators and the low levels and low titers of H2 antibodies suggest either differences in test specificity, sensitivity, or both. More recent application of the seroarcheologic model failed to confirm the proposed link of preexisting H2 antibodies with the 1890 pandemic (*32*).

In contrast, preexisting high levels of H3 antibodies among persons >85 years of age in 1968 were common findings in all serologic tests. Some investigators linked the origin of preexisting H3 antibodies to the minor 1900 pandemic (*11**,**14*), whereas others favored the 1890 pandemic (*33*). Observations from recent application of the validated H1 seroarcheologic model to published data linked preexisting H3 antibodies to the pandemic of 1890 (*32*).

We can reasonably conclude that the virus (H3) with the highest HI antibody titers and highest peak antibody prevalence (>90%) in the elderly resulted not from an epidemic (1900) but a pandemic (1890). The virus (H2) with the lowest HI antibody titers and seroprevalence (15%–29%) in the elderly is an unlikely candidate for the most severe influenza event of the late 19th century.

### Epidemiologic Findings Linking 1890 with H3

Population immunity against the shared neuraminidase (N2) antigens between the 1968 H3N2 pandemic strain and its H2N2 predecessor is believed to have contributed to the low number of deaths observed in 1968 and 1969 (*12*). However, more dramatic was the selective sharp decrease in expected excess deaths among persons born before 1893. In the 1970 wave that followed, no excess deaths occurred in persons born before 1885 (*34*). Influenza infection rates in 1968 and 1969 among persons born before 1890 were two thirds lower than among persons born after 1899 (*35*), further linking H3 with 1890.

### Unclear Evidence for H2 Recycling

No single, simple explanation has been proposed for the reported low levels of preexisting H2 antibodies before 1957. Whether these antibodies, if specific, represented cross-reactions stimulated by a related virus or by the H3 virus itself is uncertain. Evidence against specificity (or at least prevalence) of H2 antibodies is the absence of any obvious protective effect among persons >75 years of age during the 1957–1958 pandemic, which is in stark contrast to the strong correlation of prepandemic antibodies with protection in 1968 and 1969 (H3) and 1977 (H1) (*36*).

Linking H2 to 1890 and H3 to 1900 may have been a historical accident. The reports of preexisting low levels of H2 antibody in persons >75 years of age predated the H3 findings by 10 years. Thus, H2 antibodies were attributed to the 1890 pandemic, the only accepted pandemic around that period. When preexisting high levels of H3 antibody were recognized in essentially the same age cohort in 1968, the 1890 pandemic slot had already been taken.

### Lack of Evidence for H1 Recycling

Researchers have long speculated (*3*) that preexisting H1 antibody among the elderly in 1918 accounted for the well-known "W" excess death curve (*37*). Theories of special protection of the population >40 years of age compete with theories of extraordinary vulnerability of young adults. But given the continued increase in the death rate curve (albeit dampened) among those >65 years of age in 1918 (*37*) and the remarkably low death rate among those with preexisting antibodies in 1968 (H3) and 1977 (H1), evidence of H1 recycling in 1918 is not compelling. With the passage of time and the absence of sera collected from persons >40 years of age before the 1918 pandemic, the issue of H1 recycling is difficult to resolve.

H1 reappeared, of course, in 1977, but evidence suggests that the 1977 H1N1 virus reemergence was not a natural event (*38**,**39*). Transmission of the mild H1N1 for >25 years, primarily among those born after 1957, coupled with the previous natural transmission among persons born before 1956, completes the H1N1 immunologic experience of all age groups. If a natural recycling sequence ever existed, present population immunity precludes H1 as a pandemic candidate for years to come.

Solid evidence of recycling exists for a single subtype, H3, which (likely with an equine N8 neuraminidase [40]) caused the pandemic of 1890 and reemerged with the N2 neuraminidase in 1968. Thus, in the last 115 years, the influenza A virus hemagglutinin had recycled in humans at least once, after 79 years. Neuraminidase subtypes during this same period of time were N8 (1890), N1 (1918), and N2 (1957 and 1968) (*40*). No evidence of neuraminidase recycling has been seen.

## Lessons from 1976

Swine influenza virus was isolated in the United States from humans for the first time in 1974, just 2 years before the Fort Dix outbreak (*41*). Additional swine virus infections of humans were confirmed by serologic evidence and virus isolation in 1975 and 1976, with a least 1 suggested incident of person-to-person spread other than the Fort Dix outbreak. Increased recognition of swine influenza infections may have been a matter of increased surveillance, number of susceptible humans, or swine virus transmissibility. Human experimental studies (*20*) and virologic findings (*42*) suggest the latter.

Influenza virus eradication in swine was recommended by WHO in 1976 (*19*), but such action was not taken because of major biologic challenges and absence of resources. Today, even if pandemic risk were absent, the economic loss from infected poultry and mounting human illness and death are compelling reasons in themselves to place highest priority on avian influenza virus control.

### Risk Assessment Limited by Available Knowledge

The major lesson from 1976 was that increased animal-to-human transmission and major outbreaks of a novel influenza virus do not necessarily lead to pandemics, at least in the short term. However, knowledge of the Fort Dix outbreak and evidence that swine influenza virus/H3N2 reassortants could occur in pigs under conditions of natural transmission (*24*) likely would have generated concerns for years about swine influenza transmission to humans had not H1N1 virus reappeared in the human population in 1977.

Since 1976, available knowledge of the influenza A virus and its natural history has expanded greatly. Multiple experimental studies have better defined conditions for virus mutation and the creation of reassortants. Opportunities for human exposure and the current number of incidences of avian virus transmission to human are unprecedented in modern times, but in 2005, as in 1976, the precise conditions that lead to the emergence of a pandemic strain are unknown.

### Concern of Virus Recycling

In recent history, influenza virus recycling has occurred twice, once through the natural process (H3 in1968) and once likely through human negligence (H1 in 1977) (*38**,**39*). If human influenza A epidemics are restricted to 3 subtypes, as some have speculated, and if H1 and H3 are presently in circulation, then only H2 remains. The risk of H2 reemerging in humans through an act of nature is theoretical. The risk of H2 reemerging through an act of human negligence is all too real.

In the published report of the April 7, 1976, WHO meeting of international experts, the final paragraph urged extreme caution in developing live vaccines from A/New Jersey strains (H1N1) because of the possible danger of spread to susceptible human or animal hosts (*19*). That paragraph was written specifically to respond to reports that several investigators outside Western Europe had plans to develop and test such vaccines. One year later, an H1N1 virus, identical to the laboratory strain from1950–1951, swept the world.

In an incident earlier this year, H2N2 virus was accidentally distributed in proficiency testing panels to laboratories in 18 countries. Recognizing the potential danger, CDC and WHO issued a health advisory on April 13, 2005, to destroy all such samples and followed on May 3 with recommendations to increase biosafety levels for H2N2. Laboratory containment of H2N2 strains is crucial. No one born since 1968, including many laboratory staff, is immune. The level of compliance with these biosafety recommendations in all areas of the world is unknown. Focusing on the theoretical risk for natural H2 emergence and ignoring the real risk in our own laboratories would be tragic.

### Risk Assessment Separate from Risk Management

Internationally, influenza risk assessment and risk management are separate functions. WHO makes risk assessments in the form of annual recommendations on influenza vaccine composition. Nations may elect to accept WHO findings and recommendations or to have their own risk assessment bodies that incorporate WHO findings. Risk management, on the other hand, is the exclusive responsibility of national governments. Independent expert bodies may make recommendations, but risk management ultimately is a political process, performed and funded by federal and state governments.

Nationally, risk assessment should also be a separate scientific function, free from influence by perceived risk-management resource constraints, organizational capacities, or political aspirations. Pandemic risk management, itself an uncertain art, must independently weigh ongoing risk-assessment findings in the context of actions that best serve national and international interests.
